# Editorial: Influence of mutations, stresses and post-translational modifications on the structure and function of heat shock proteins and their relationship with various diseases

**DOI:** 10.3389/fmolb.2023.1310272

**Published:** 2023-10-20

**Authors:** Alok Kumar Panda, Ashis Biswas

**Affiliations:** ^1^ Environmental Science Laboratory, School of Applied Sciences, KIIT Deemed to be University, Bhubaneswar, India; ^2^ School of Basic Sciences, Indian Institute of Technology Bhubaneswar, Bhubaneswar, India

**Keywords:** heat shock proteins, stress client proteins, holdase, chaperone function, post-translational modifications, mutations, diseases

Heat shock proteins (Hsps) are molecular chaperones induced under stress conditions such as oxidative, heat, acidic, *etc.*, and prevent the aggregation/mis-folding of various client proteins that are prone to partial denaturation and aid in the folding of the nascent proteins (Haslbeck and Vierling, 2015). Heat shock proteins are classified into large and small heat shock proteins based on their monomeric molecular weight. Large heat shock proteins (LHsps) help in the protein folding mostly via ATP dependent manner (Gusev et al., 2002). However, small heat shock proteins (sHsps) generally function in an ATP independent manner and are regarded as the first line of defense against stress. They exhibit holdase function (Basha et al., 2012). Both large and small heat shock proteins are found in almost all organisms and are characterized by their signature conserved domain. Mutations, stresses, and post-translational modifications (PTMs) of Hsps are implicated in various diseases that often modulate the structure, substrate binding capability, subunit exchange dynamics, and chaperone function of Hsps. Therefore, understanding the structure-function relationship of Hsps with different diseases will provide new avenues for building better therapeutics and drugs by targeting the Hsps. Investigating the role of structural and functional modulations of Hsps (due to mutations, stresses, and PTMs) in diseases is multidisciplinary in nature. It involves the intervention from the areas of molecular biology, microbiology, biophysics, biophysical chemistry, cell biology, biochemistry, and protein engineering.

The objective of this Frontiers Research Topic was to bring together a series of original articles, and reviews, and other article types that address the structural-functional role of sHsps in diseases. We aim to collect studies that outline the role of mutations, different stresses, and PTMs on the structure and function of Hsps and their relationship to the disease. This will expand our knowledge of the relationship between structure-function modulations of Hsps with diseases and will be a steppingstone towards the generation of new therapeutics.

For this Research Topic we received many submissions from which 4 articles by 16 authors across the globe were published. The articles included not only original research paper but also contained reviews.

Piplani et al. reviewed the implications of mutations and posttranslational modifications in heat shock proteins on human diseases and health. In this review, the authors have explained the role of heat shock proteins in maintaining and restoring cellular proteostasis by preventing the formation of the protein aggregates. The review also explains the effect of posttranslational modifications and mutations on the regulatory mechanisms of heat shock proteins. It also illuminates the implications of these mutations and modifications on the conformations of the heat shock proteins which leads to several disorders and diseases.

Perna et al. have revealed that sacsin, a protein linked to neurodegeneration contains alpha fold structure like that of Hsp90 which also harbors the key residues for ATPase activity. In this study based on the molecular dynamic simulation the authors hypothesize that not all ATP competitive inhibitors of Hsp90 may bind to sacsin. Their analysis also supports the fact that sacsin’s function is ATP driven.

Serricchio and Bütikofer have shown that a conserved mitochondrial chaperone-protease complex plays a vital role in protein homeostasis. In this work the authors have shown that TbSlp2 protein from *Trypanosoma brucei* complexes with the metalloprotease TbYme1 form large mitochondrial-protein complex. They have further shown that TbYme1 plays a vital role under heat stress, which lead to the loss of viability of the organism.

Lastly research work done by Kaku and Rothstein reveals Fas Apoptosis Inhibitory Molecule (FAIM) as a novel molecule that plays a role in maintaining cellular viability and homeostasis under heat and oxidative stress. The work also suggests that FAIM interferes in the aggregation process of stress induced proteins, due to which the amount of protein aggregates decreases in the cell lines. This work reports a novel molecule FAIM which protects the cell against stress and viability loss due to protein aggregation.

Altogether, we hope that this Research Topic of original research articles and review for this Research Topic provides new perspectives and insights into the wide range of connections between “structural and functional modulations of HSPs due to mutations, post-translational modifications and stresses” with different important human diseases.
